# The dye-sensitized solar cell database

**DOI:** 10.1186/s13321-018-0272-0

**Published:** 2018-04-03

**Authors:** Vishwesh Venkatraman, Rajesh Raju, Solon P. Oikonomopoulos, Bjørn K. Alsberg

**Affiliations:** Department of Chemistry, NTNU, Høgskoleringen, 7491 Trondheim, Norway

**Keywords:** Dye sensitized solar cells, Database

## Abstract

**Background:**

Dye-sensitized solar cells (DSSCs) have garnered a lot of attention in recent years. The solar energy to power conversion efficiency of a DSSC is influenced by various components of the cell such as the dye, electrolyte, electrodes and additives among others leading to varying experimental configurations. A large number of metal-based and metal-free dye sensitizers have now been reported and tools using such data to indicate new directions for design and development are on the rise.

**Description:**

DSSCDB, the first of its kind dye-sensitized solar cell database, aims to provide users with up-to-date information from publications on the molecular structures of the dyes, experimental details and reported measurements (efficiencies and spectral properties) and thereby facilitate a comprehensive and critical evaluation of the data. Currently, the DSSCDB contains over 4000 experimental observations spanning multiple dye classes such as triphenylamines, carbazoles, coumarins, phenothiazines, ruthenium and porphyrins.

**Conclusion:**

The DSSCDB offers a web-based, comprehensive source of property data for dye sensitized solar cells. Access to the database is available through the following URL: www.dyedb.com.

**Electronic supplementary material:**

The online version of this article (10.1186/s13321-018-0272-0) contains supplementary material, which is available to authorized users.

## Background

**Fig. 1:**
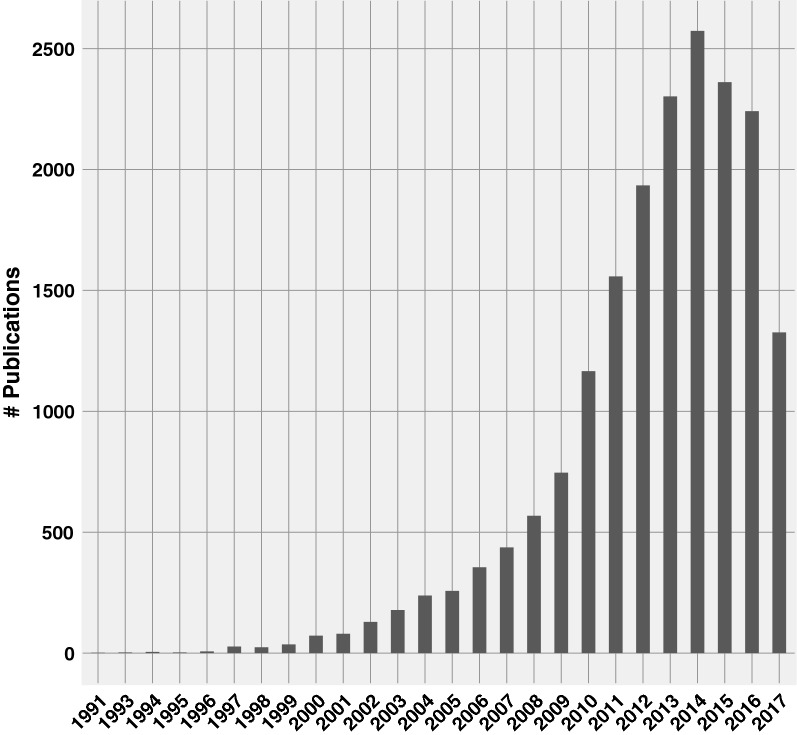
Literature growth of dye sensitized solar cells. The graph was produced by performing a Web of Science search for the keywords “dye sensitized solar cells” and restricting the search to articles in English. In addition subject areas such as mechanics, toxicology, pharmacology and educational research were also excluded to yield around 18445 records

With renewable energy-based systems gaining momentum, there has been intense focus on harnessing energy from the sun while making it useable and cost-effective. Solar cell technologies that convert sunlight into electricity include those based on silicon, polymer, quantum dots, dye-sensitized and more recently, perovskites. Among these dye-sensitized solar cells (DSSCs) also known as Grätzel cells have received a lot of attention [1, 2]. In recent years, this field has seen a dramatic increase in published research. An ISI Web of Knowledge search for the term “dye-sensitized solar cells” yielded more than 18,000 articles spanning years 1991–2017 (see Fig. 1) with a significant proportion published in the last 5–10 years.

The DSSC typically consists of a monolayer of a photosensitive dye that is adsorbed on a mesoporous oxide layer (such as $\hbox {TiO}_{2}$, ZnO or $\hbox {SnO}_{2}$) that is deposited on a transparent conductive glass substrate, a redox electrolyte (iodide or cobalt-based) and a platinized counter electrode. On excitation (absorption of incoming light), electrons from the dye diffuse (flow of current) through the semiconductor and move on to the back collecting electrode. Dye regeneration takes place through electron donation from the electrolyte aided by the catalyst in the counter electrode. The modular architecture of the DSSC thus enables functions such as electron transport, light absorption and hole transport to be handled separately [1, 2].

Although DSSC efficiencies have been improving, the pace of these improvements has been somewhat slow, with an increase of only $\thicksim 6\%$ [3] from a value of 7.1% in 1991 [4]. The variations in the power conversion efficiencies (PCE) for different DSSCs can be attributed to changes in the cell architecture and fabrication [5–8]. While a significant amount of these efforts have been devoted to molecular engineering of the dye sensitizer [9–13], others have focused on the optimization of the electrodes [14, 15] and electrolytes [16–18] along with factors such as the concentrations of the solvent baths during sensitization [19], and the size and thickness of photoanodes [8, 20, 21].

The DSSC efficiency is influenced by a number of components/parameters. Other than the dye one can add a cosensitizer to account for the higher wavelength regions, use agents like chenodeoxycholic acid (CDCA) to reduce aggregation and also change the electrolyte from iodide to cobalt which has often resulted in increased PCEs. Other factors such as the dye bath and concentrations also have an impact on the PCEs. Modifications to the structure of the dye sensitizer in particular have been found to be the most widely applied method to improve device efficiencies. Given that, by introducing systematic variations of the substituent groups in the dye can improve the light harvesting and electron injection capabilities among other properties, various classes of dyes (metal-free and inorganic-based) have been investigated ranging from coumarins, carbazoles, indolines, triphenylamines [10], phenothiazines [9], fulvalenes [22] to ruthenium [23] and porphyrin-based [24] sensitizers.

Dye materials discovery has been largely based on serendipity or iterative chemical substitution. Given the demand for methodologies that can accelerate the design of molecular materials with tailored properties, cheminformatics (or materials informatics) based frameworks for high-throughput screening of candidate structures have been proposed: dyes [25], solid state metal oxide photovoltaic cells [26] and organic photovoltaics [27, 28]. With a view to understanding how structural/chemical modifications impact the solar cell performances, recent efforts have focused on creating quantitative structure property relationships [26, 29–36] that establish a mathematical relationship between various molecular structure descriptors and a solar cell property of interest such as the PCE. The models produced in this process have been further used to direct the search for promising dyes/photvoltaic materials that satisfy desirable criteria [26, 37–39]. Informatics approaches have also been recently applied to the identification of suitable photocathode materials [40] and solid state electrolytes [41].

Recently, a number of data repositories such as the Materials Project [42], Khazana [43], the Harvard Organic Photovoltaic Dataset [44], and the Open Spectral Database [45] have emerged that facilitate the discovery of qualitative/quantitative rules, which can be used to guide materials design. Here, we report the Dye Sensitized Solar Cell Database (DSSCDB) consisting of experimental results compiled from the literature. The database is intended as a central repository for sharing photovoltaic performance related data and should be of broad interest to scientists in photovoltaics, quantum chemistry, chemometrics and related disciplines. Search tools have been implemented with both text and structure-based functionalities.

## Description and utility

Information regarding the dyes was manually retrieved from journal articles obtained using keyword (“dye sensitized solar cells”, “triphenylamines” etc.) searches on the ISI Web of Knowledge. For each dye, the following data has been recorded:

1. DOI: the digital object identifier for the referenced article
2. Performance parameters: open circuit voltage ($V_{oc}$ in mV), short circuit current ($J_{sc}$ in $\hbox {mA/cm}^{2}$), fill factor (FF), power conversion efficiency (PCE)
3. Electrolyte: nature of redox electrolyte (iodide/cobalt-based) and the composition
4. Active area of the cell (in $\hbox {cm}^{2}$)
5. Coadsorbents such as chenodeoxycholic acid (CDCA) and their concentration if used
6. Cosensitizer if used
7. Semiconductor: the kind of semiconductor layer ($\hbox {TiO}_{2}$, ZnO etc.) used and their thickness (in μm) along with any scattering used
8. Dye loading: the amount of dye adsorbed on the semiconductor film (in $\hbox {nmol/cm}^{2}$)
9. Exposure time: the sensitizer adsorption time
10. Solar simulator conditions such as the light intensity (typically AM 1.5G, 100 $\hbox {mW/cm}^{2}$) used
11. DSSC comments: general information relating to the type of the DSSC (solid state, p-type), the dye bath used and other details such as the surface treatment for the semiconductor or electrodes
12. SMILES, InChi: SMILES notation for the chemical structure and the corresponding IUPAC International Chemical Identifier (InChi) key
13. The absorption and emission maxima and the solvent used in the experiment
14. Dye class: to indicate the type of the donors or specific chemical groups in order to enable a keyword-based search.

**Fig. 2:**
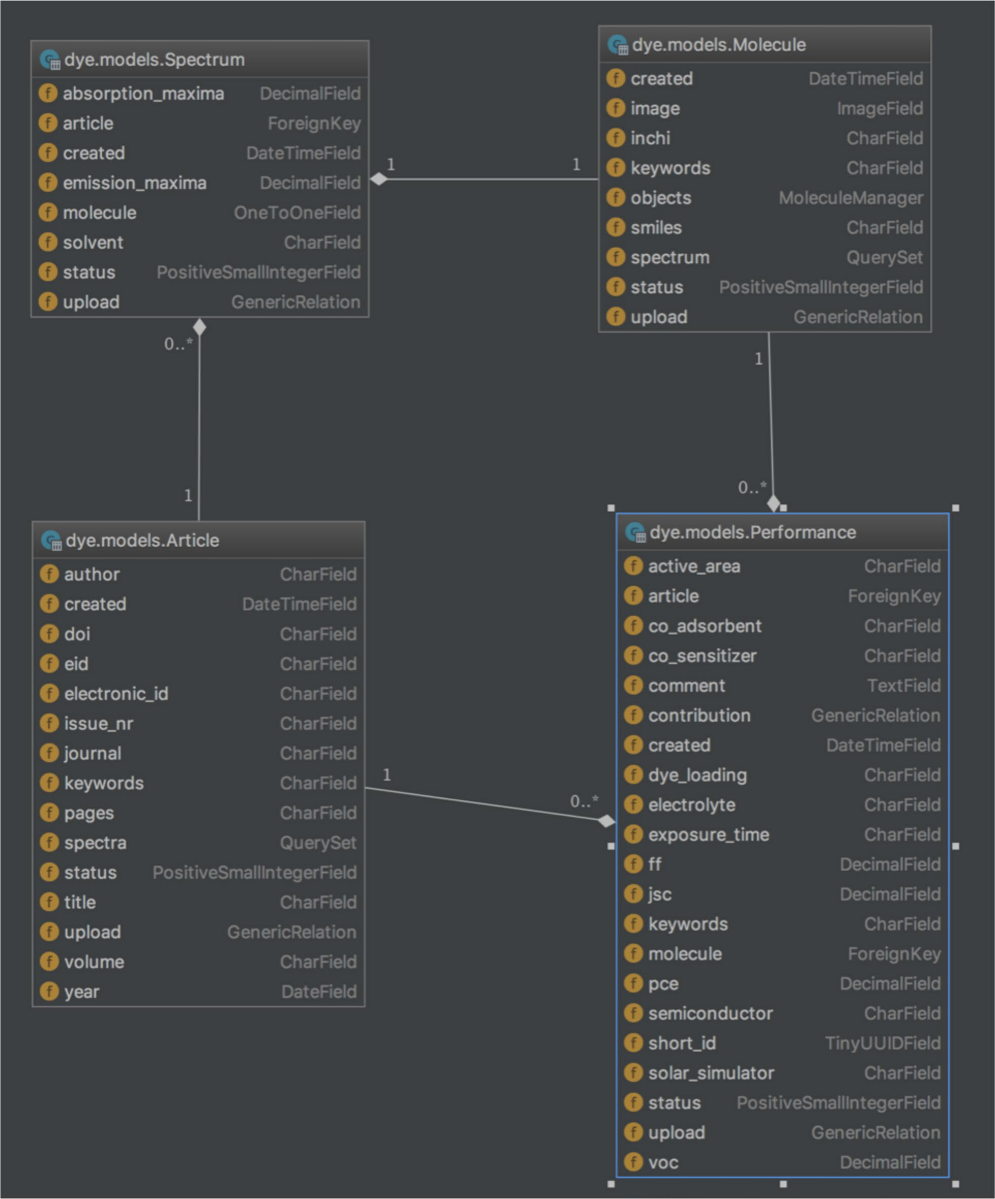
Database structure. Schematic representation of the DSSCDB

The database is centred around 4 main tables (see Fig. 2) reflecting the aforementioned details. During the data collection, articles without a valid DOI and those with incomplete performance data were excluded. The 2D structures of the dyes were drawn using various molecular drawing software. For cases where the chemical names were available, the SMILES formats were generated using OPSIN [46], failing which the structures were drawn by hand. Corresponding InChi keys were then generated using OpenBabel [47, 48]. Images of the structures have been generated using the Indigo Toolkit [49]. The web interface has been designed using the Django 1.10 MVC framework (https://www.djangoproject.com) and connected to a PostgresSQL [50] database and hosted on the Amazon Cloud Platform. The package manager Conda 4.3.6 (https://www.conda.io) was used to include RD-Kit 2017.03.1 [51] and PyBel [52] support. The Docker platform (https://www.docker.com/) was additionally used to facilitate continuous development and ease of deployment.

### Search and retrieval

**Fig. 3:**
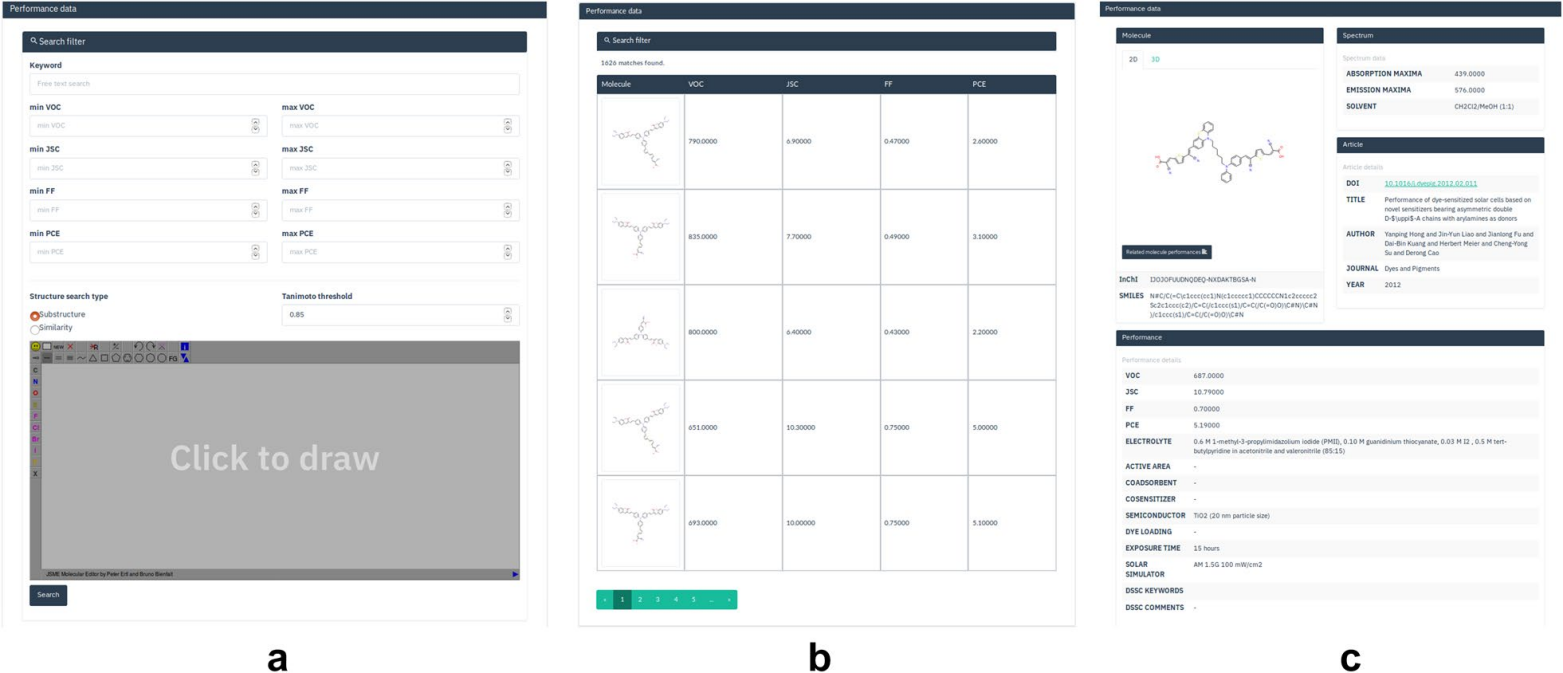
Search and retreival. **a** Searching the database can be carried out using a text-based query, a range-based search or alternatively based on a substructure which can created using the molecule editor. **b** Search results based on searching for “triphenylamine”. **c** Additional details pertaining to a given row can be obtained by clicking on the row

The entire database can downloaded as a csv file which contains SMILES, InChi, performance data, experimental conditions, chemical scaffold type, and links to the articles from which the data was sourced. Data can be retrieved using either a range search based on options such as the PCE, FF, $J_{sc}$, $V_{oc}$ or alternatively performing a free text search for specific dye classes (see Fig. 3). A search for triphenylamines for instance yields more than 1600 results. For each structure, the class of the dye (triphenylamine, coumarin etc.) have been recorded which facilitates the search for specific dyes. Structure-based searches (drawn using the JSME Molecular Editor [53]) can be carried out using either fingerprint-based Tanimoto similarity or SMARTS-based substructure matching, functions for which are available in the Pybel library [52]. The results are presented in a table with details for each entry provided in a separate page. The structure can be visualized as a 2D diagram or alternatively in three dimensions using the WebGL based 3dmol library [54]. For cases, where multiple results are available for the same structure, links to related entries are provided.

### Uploading data

**Fig. 4:**
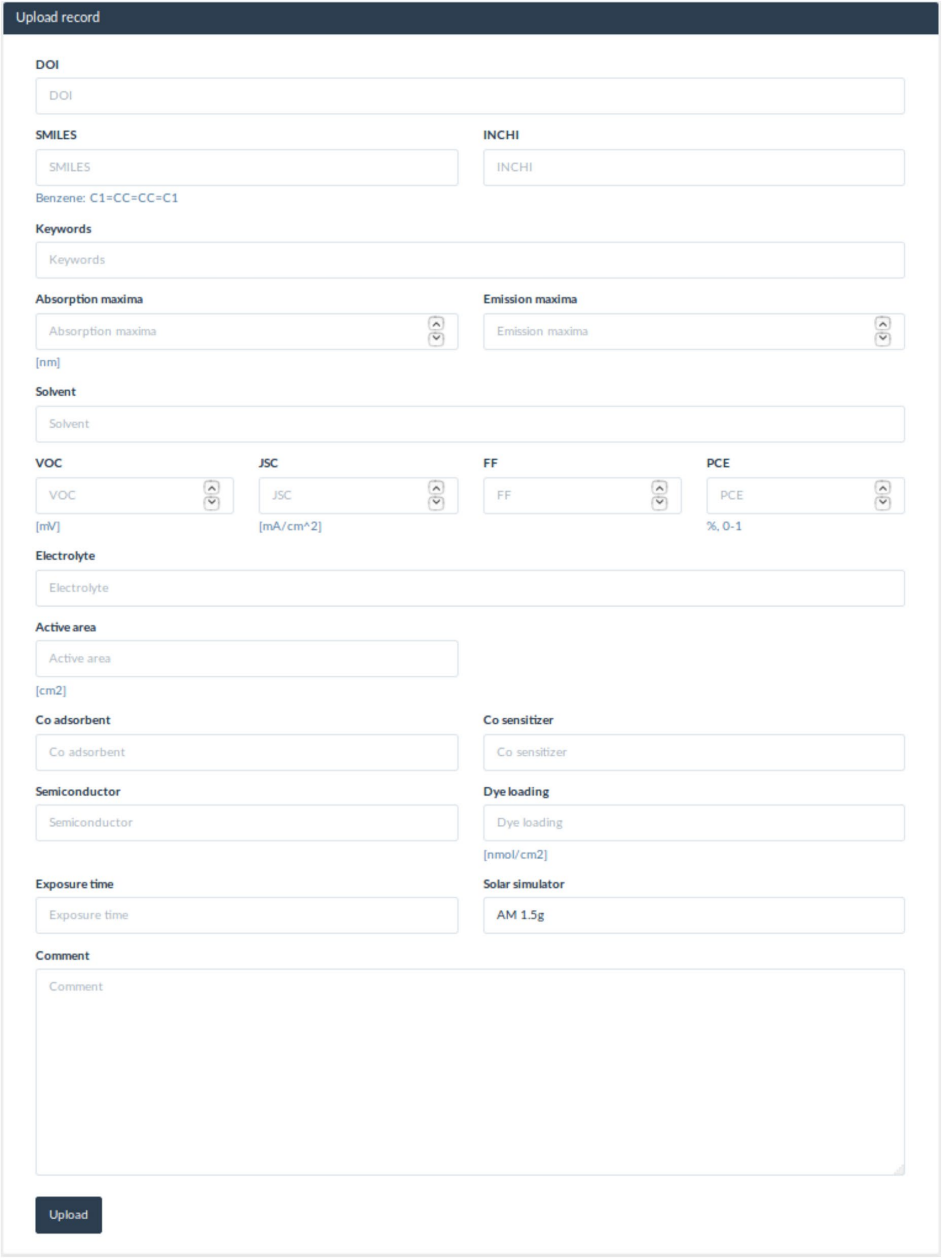
Single data entry. The web interface to enter single records

To contribute to the DSSCDB, users are firstly required to register themselves. Data for the dyes can be entered in two ways. While single entries can be entered using the web interface (see Fig. 4), an Excel file containing the columns to be filled can be used for multiple entries (template available as part of the supplementary information). The uploaders are required to provide the molecular structure information (valid SMILES and InChi codes), performance data, experimental conditions, chemical scaffold (coumarin, porphyrin etc.) and the digital object identifier for the article from which the data was collected. In order to ensure the validity of the data entered, a verification step has been added whereby the database administrators can confirm the authenticity of the data.

### Data summary

**Fig. 5:**
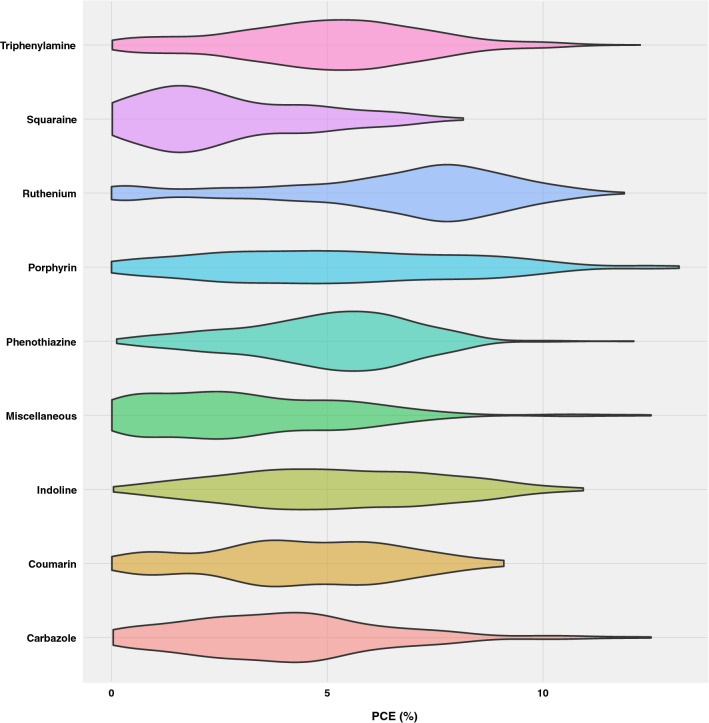
Compound classes and efficiency ranges in the DSSCDB. Violin plots showing the distribution of the efficiencies. Values in the wider parts of the violin are more probable than those in narrower regions. The “Miscellaneous” class includes dyes based on donors such as perylene, azobenzene, anthracene, fulvalene, imidazole and julolidine

The database currently holds over 4000 experimental results spanning a diverse set of metal-free and metal complex dyes. Metal-free sensitizers are dominant in the database among which triphenylamines form the most prominent class. Figure 5 offers a visual summary of the reported efficiencies for the different dye classes.

**Fig. 6:**
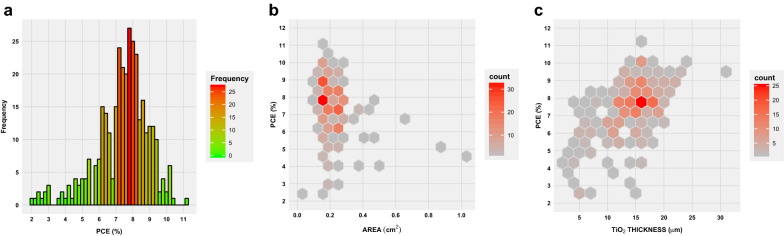
Summary of efficiencies for the reference dye Ruthenium N719. **a** Chart shows the distribution of the reported efficiencies (over 300 cases) for N719. **b** 2D histogram of the PCE versus the active area of the device, **c** 2D histogram of the PCE versus the thickness of the $\hbox {TiO}_{2}$ layer used

In many articles, device performances based on new dyes being tested are compared with what has been frequently referred to as a benchmark/reference dye which is typically a Ruthenium dye commonly known as N719. Querying “N719” on the DSSCDB yields around 329 results collated from over 250 articles. The reported efficiencies which range between 2 and 11% for these records are summarized in Fig. 6. Active areas and the thickness of the semiconductor layer are often the most varied parameters in many studies. The impact of this variation can be studied in terms of 2D histograms shown in Fig. 6b, c respectively. Here, the hexagons coloured in shades of red indicate the count of the combinations. For instance, it is seen that areas around 0.16–0.20 $\hbox {cm}^{2}$ have average efficiencies of around 7–8% in more than 30 cases, while a thickness of around 16–18 μm for the $\hbox {TiO}_{2}$ layer yielding similar efficiencies is seen for 25 or more cases. However, as can be seen from the plots, there are also other settings that can lead to lower or higher values.

Given that, such subtle changes can lead to an increase or decrease in the efficiency, how much of this improvement is real and significant is difficult to ascertain. From a data analysis perspective, particularly for QSPR modelling, identifying observations with comparable experimental criteria poses significant challenges. The need for proper characterization and standardization of measurement protocols has been highlighted in a number of publications [55–57]. Although seeking confirmation from independent certification laboratories, very few publications report such steps. We hope that in due course, the best-practice characterization procedures can be adopted.

## Conclusions

A first of its kind database for dye sensitized solar cells is described. The repository offers an at a glance summary of the experimental conditions that led to the reported efficiencies and properties and is expected to be an important asset for scientists and researchers in the field of photovoltaics and associated fields. It is our hope that the database can be used to extend knowledge and stimulate new directions for design and development of photovoltaic materials. We anticipate expanding and updating this collection over time and further extend the database to dyes used in other fields such as light emitting diodes, food products etc.

## Additional files

**Additional file 1:** Full search criteria used on the Web of Science.

**Additional file 2:** Excel file template for uploading data.
